# Trends and Risk Factors Associated With Stroke Recurrence in China, 2007-2018

**DOI:** 10.1001/jamanetworkopen.2022.16341

**Published:** 2022-06-15

**Authors:** Jie Xu, Xing Zhang, Aoming Jin, Yuesong Pan, Zixiao Li, Xia Meng, Yongjun Wang

**Affiliations:** 1 Department of Neurology, Beijing Tiantan Hospital, Capital Medical University, Beijing, China; 2 China National Clinical Research Center for Neurological Diseases, Beijing, China; 3 Advanced Innovation Center for Human Brain Protection, Capital Medical University, Beijing, China; 4 Research Unit of Artificial Intelligence in Cerebrovascular Disease, Chinese Academy of Medical Sciences, 2019RU018, Beijing, China; 5 Center for Excellence in Brain Science and Intelligence Technology, Chinese Academy of Sciences, Shanghai, China

## Abstract

**Importance:**

Recurrent stroke rates have decreased substantially in Western countries. However, data on changes in stroke recurrence and risk factor patterns in China are limited.

**Objective:**

To systematically assess stroke recurrence trends by evaluating temporal improvement in guideline-recommended secondary prevention treatment performance and changes in risk factor patterns over 10 years in China.

**Design, Setting, and Participants:**

This post hoc cohort study was conducted based on data from the China National Stroke Registries (CNSRs, comprising 3 phases, I-III, from 2007-2018). Participants were patients with ischemic stroke who were enrolled in CNSR I or III within 7 days of symptom onset. Data were analyzed from September through November 2021.

**Exposures:**

Vascular risk factors included current smoking, alcohol consumption, hypertension, diabetes, coronary artery disease, atrial fibrillation, and low-density lipoprotein cholesterol (LDL-C) levels.

**Main Outcomes and Measures:**

The cumulative incidence rates of stroke recurrence at 3, 6, and 12 months were calculated, and the performance of guideline-based secondary prevention treatments was investigated at each visit in CNSR I (2007-2008) and III (2015-2018). Logistic regression models were used to evaluate changes in risk factor patterns for stroke recurrence based on data from CNSR I and III.

**Results:**

A total of 10 952 patients with ischemic stroke from CNSR I (6740 [61.5%] men; median [IQR] age, 67 [57-75] years) and 10 348 patients with ischemic stroke from CNSR III (7128 [68.9%] men; median [IQR] age, 63 [54-70] years) were selected. Over 10 years, the adjusted cumulative incidence of recurrent stroke within 12 months decreased from 15.5% (95% CI, 14.8%-16.2%) to 12.5% (95% CI, 11.9%-13.1%) (P < .001). Factors associated with increased risk of stroke in CNSR I that were still associated after 10 years included age per 10 years (CNSR I: odds ratio [OR], 1.24; 95% CI, 1.18-1.31; CNSR III: OR, 1.08; 95% CI, 1.01-1.15), prior stroke (CNSR I: OR, 1.62; 95% CI, 1.45-1.82; CNSR III: OR, 1.66; 95% CI, 1.44-1.92), coronary heart disease (CNSR I: OR, 1.21; 95% CI, 1.04-1.40; CNSR III: OR, 1.23; 95% CI, 1.02-1.49), and LDL-C level per 10 mg/dL (0.259 mmol/L) (CNSR I: OR, 1.02; 95% CI, 1.01-1.04; CNSR III: OR, 1.02; 95% CI, 1.00-1.03), whereas atrial fibrillation (CNSR I: OR, 1.51; 95% CI, 1.26-1.81; CNSR III: OR, 0.95; 95% CI, 0.74-1.23) was no longer an independent risk factor in 2015 to 2018.

**Conclusions and Relevance:**

This study found that stroke recurrence rate in China decreased significantly, but approximately 12.5% of patients still experienced stroke recurrence within 12 months. These findings suggest that more intensive control of traditional risk factors, including LDL-C levels, may be needed to further reduce stroke recurrence.

## Key Points

**Question:**

What are the trends in stroke recurrence rates and factors associated with recurrent stroke in China, and are secondary preventive treatments used?

**Findings:**

This cohort study of 10 952 patients with ischemic stroke in 2007 to 2008 and 10 348 patients with ischemic stroke in 2015 to 2018 found a significant decrease in stroke recurrence rates in China in the past decade. Higher low-density lipoprotein cholesterol levels, as well as age, prior stroke, and coronary heart disease, were associated with recurrence despite advances in secondary prevention treatments.

**Meaning:**

These findings suggest that more intensive control of traditional risk factors may be needed to further reduce stroke recurrence.

## Teaser

This cohort study investigates the association of treatment and risk factors with stroke recurrence and changes in risk factor patterns over 10 years in China.

## Introduction

Rates of recurrent stroke have decreased substantially in Western countries in the past decades,^1,2,3,4^ largely associated with advancements in the use of guideline-recommended secondary preventive measures and control of vascular risk factors.^5,6^ However, little is known about the contemporary trends of stroke recurrence in China. Clinical practice in China recently attached great importance to acute management and secondary prevention measures after the occurrence of acute ischemic stroke (IS) as recommended by guidelines^7,8^; however, stroke remains a substantial challenge faced by the Chinese population.^9,10^ Despite improvements in evidence-based secondary prevention measures targeted at controlling traditional risk factors, patients with IS still appear to be at a residual risk of stroke recurrence.^11,12,13^ This risk is likely associated with suboptimal control of traditional risk factors or lack of recognition and management of potential novel risk factors.^14,15^ Nevertheless, few studies have investigated whether traditional risk factors,^16,17,18,19,20^ including hypertension, diabetes, hyperlipidemia, and atrial fibrillation, are still associated with recurrence risk, and few studies have evaluated dynamic transitions of vascular risk factor patterns for stroke recurrence among the Chinese population.

Objective and comparable populations and data are required to assess changes in stroke recurrence rates and risk factor patterns systematically and accurately. The China National Stroke Registries (CNSRs) are national, hospital-based, prospective stroke registries comprising 3 phases spanning 10 years. CNSRs I and III were conducted between September 2007 and August 2008 and August 2015 and March 2018, respectively. These CNSR phases provided an opportunity to assess trends and transitions in stroke recurrence and risk factor patterns over 10 years.

## Methods

The design and rationale of the CNSRs used in this cohort study were approved by the ethics committee of Beijing Tiantan Hospital and participating hospitals. Written informed consent was obtained from participants or their legally authorized representatives. CNSR ethic committee approval and satisfaction of informed consent requirement extended to this study. The study adhered to the Strengthening the Reporting of Observational Studies in Epidemiology (STROBE) reporting guideline. The data supporting the findings of this study are available from the corresponding author upon reasonable request after clearance by the ethics committee.

### Registry Characteristics and Study Population

CNSRs are nationwide prospective registries of patients with acute cerebrovascular events used to evaluate characteristics of patients who experienced strokes. CNSR I recruited 21 902 patients with acute cerebrovascular events within 14 days of the index event from 132 hospitals that cover all 27 provinces and municipalities in China between September 2007 and August 2008. CNSR III recruited 15 166 patients with IS or transient ischemic attack (TIA) within 7 days from symptom onset to enrolment from 201 hospitals that cover 22 provinces and 4 municipalities in China between August 2015 and March 2018. Detailed study design and patient characteristics of CNSR I^21^ and CNSR III^22^ have been published previously.

This study was based on data from CNSR I and III. Information on hospitals for study patients are presented in the eMethods in the Supplement. Among 33 hospitals included in CNSR I and CNSR II, 30 hospitals (90.9%) were tertiary facilities. Patients with IS who were enrolled within 7 days after symptom onset were selected from CNSR I and III in our analysis, and stroke was confirmed using brain computed tomography or magnetic resonance imaging.

### Data Acquisition

Baseline data were collected by trained research coordinators at each institute who followed a standard data-collection protocol. These data included sex, age, education level, body mass index (calculated as weight in kilograms divided by height in meters squared), medical history (ie, prior stroke, coronary heart disease, hypertension, diabetes, and atrial fibrillation), smoking and drinking status, stroke profile (National Institute of Health Stroke Scale [NIHSS] score at admission), in-hospital therapy, discharge status, and lipid levels. Additional information on data collection and variable definitions are described in CNSR I^21^ and III^22^ protocols.

### Outcomes

Patients were interviewed face to face at 3 months and contacted via telephone by trained research coordinators at 6 and 12 months after enrolment. Information on cardiovascular and cerebrovascular events and compliance with recommended secondary preventive medication was collected. Confirmation of vascular events was sought from the treating hospital, and suspected recurrent cerebrovascular events without hospitalization were judged by an independent end point judgment committee.

The study outcome was a new stroke (including ischemic and hemorrhagic strokes) during follow-up. This was defined as an aggravated primary neurological deficit (ie, NIHSS score increased by ≥4 points), a new neurological deficit lasting longer than 24 hours, new brain lesions confirmed using imaging (computed tomography or magnetic resonance imaging), expansion of the original lesions, or rehospitalization with a diagnosis of IS, intracerebral hemorrhage, or subarachnoid hemorrhage.

### Definition of Variables

Definitions of vascular risk factors (including current smoking, current drinking, history of stroke, hypertension, diabetes, coronary artery disease, and atrial fibrillation) and medicine indications remained unchanged. We evaluated the performance of 5 evidence-based secondary preventive interventions (ie, antiplatelets, statins, anticoagulants, antihypertensives, and hypoglycemics). Detailed definitions of risk factors and medicine indications and a list of medications in each category are presented in the eMethods in the Supplement. Medicine performance at each visit was defined as the proportion of patients who received measures for which they were eligible.

### Statistical Analysis

Categorical variables were presented as numbers and percentages and continuous variables as medians with IQRs if nonnormally distributed. Baseline characteristics of the study population were compared using χ^2^ statistics for categorical variables and the Mann-Whitney U test for continuous variables. Among 10 952 participants in CNSR I, 1272 individuals (11.6%) had missing data on LDL-C levels; therefore, we imputed these missing data with multiple imputations using a fully conditional specification approach based on baseline characteristics.^23^

Cumulative rates of stroke recurrence at 3, 6, and 12 months were calculated, and adjusted rates were calculated by age, sex, and NIHSS score on admission. To evaluate changes in the performance of guideline-based secondary preventive medicines over the past decade, usage rates of 5 secondary preventive medications (ie, antiplatelets, statins, anticoagulants, antihypertensives, and hypoglycemics) at each visit (during hospitalization, at discharge, and 3 and 12 months later) for which patients were eligible were calculated in the 2007 to 2008 and 2015 to 2018 sets. To assess trends in risk factor patterns in 2007 to 2008 and 2015 to 2018, we applied multivariable logistic regression models to examine adjusted risk factors associated with stroke recurrence based on data from CNSR I (2007-2008) and III (2015-2018). Furthermore, data from 2 registries were combined into a whole-analysis data set. Study period (ie, CNSR I or CNSR III) and interaction terms of study period and covariates were additionally included in the logistic regression model. A test for interaction term was used to evaluate the interaction of study period in the associations of risk factors with stroke recurrence. Given that nearly 12% of patients in CNSR I had missing data on LDL-C levels, a sensitivity analysis was performed for CNSR I by repeating the analysis using imputed data by a fully conditional specification approach. Owing to the heterogeneity of hospitals included in CNSR I and III, we also performed a sensitivity analysis on patients from 33 hospitals that participated in CNSR I and III.

Given the competing risk for death, we performed logistic regression models excluding individuals who died within 1 year after stroke onset in the sensitivity analysis. Odds ratios (ORs) with 95% CIs were calculated. A 2-sided *P* value < .05 was recognized as statistically significant. SAS statistical software version 9.4 (SAS Institute) was used for all statistical analyses. Data were analyzed from September through November 2021.

## Results

### Baseline Characteristics

After excluding patients diagnosed with TIA and those enrolled more than 7 days after symptom onset (eFigure 1 in the Supplement), 10 952 patients with IS from CNSR I (6740 [61.5%] men; median [IQR] age, 67 [57-75] years) and 10 348 patients with IS from CNSR III (7128 [68.9%] men; median [IQR] age, 63 [54-70] years) were included. Baseline clinical characteristics of the study population by period are shown in the Table. For CNSR I vs CNSR III, there were differences in age (median [IQR], 67 [57-75] years vs 63 [54-70] years) and sex (6740 [61.5%] men vs 7128 [68.9%] men), as well as education level, current smoking and drinking status, history of stroke, diabetes, coronary heart disease, time between onset and admission, NIHSS score on admission, and triglyceride, high-density lipid cholesterol, and low-density lipid cholesterol (LDL-C) levels.

**Table.** Baseline Characteristics of Study Population

Characteristic	Patients, No. (%)		*P* value
	CNSR I (N = 10 952)	CNSR III (N = 10 348)	
Age, median (IQR), y	67 (57-75)	63 (54-70)	<.001
Men	6740 (61.5)	7128 (68.9)	<.001
Education level			
≤Elementary school	5028 (45.9)	2852 (27.6)	<.001
Middle school	2813 (25.7)	3030 (29.3)	
≥High school	3111 (28.4)	2957 (28.6)	
Unknown	NA	1509 (14.6)	
Current smoking	2912 (26.6)	3328 (32.2)	<.001
Current drinking	2974 (27.2)	1735 (16.8)	<.001
Medical history			
Prior stroke	3762 (34.3)	2316 (22.4)	<.001
Hypertension	7017 (64.1)	6533 (63.1)	.16
Diabetes	2367 (21.6)	2498 (24.1)	<.001
Coronary heart disease	1613 (14.7)	1117 (10.8)	<.001
Atrial fibrillation	848 (7.7)	753 (7.3)	.20
Time from onset to admission, median (IQR), d	1 (0-2)	1 (0-2)	<.001
NIHSS score on admission, median (IQR)	5 (2-9)	3 (2-6)	<.001
Lipid level, median (IQR), mg/dL^a^			
Triglycerides	124.78 (89.38-182.30)	121.24 (91.15-167.26)	<.001
HDL-C	45.17 (37.84-54.44)	41.31 (34.75-49.42)	<.001
LDL-C	108.11 (86.10-131.27)	90.35 (67.57-116.22)	<.001
Thrombolytic therapy	420 (3.8)	967 (9.3)	<.001

Abbreviations: CNSR, China National Stroke Registry; HDL-C, high-density lipoprotein cholesterol; LDL-C, low-density lipoprotein cholesterol; NA, not applicable; NIHSS, National Institutes of Health Stroke Scale.

SI conversion factors: To convert HDL-C and LDL-C to mmol/L, multiply by 0.0259; triglycerides to mmol/L, multiply by 0.0113.

^a^ The number of patients with triglyceride, HDL-C, and LDL-C levels measured in CNSR I was 9840, 9725, and 9680, respectively.

### Trends in Recurrence Rate

**Figure 1.**
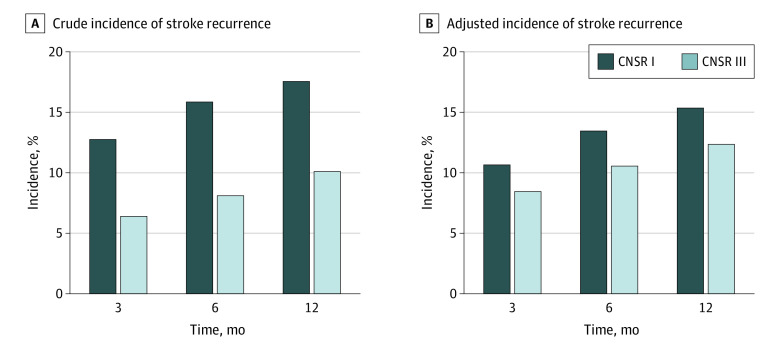
Cumulative Incidence of Stroke Recurrence Incidence rates were collected at 3, 6, and 12 months after discharge in China National Stroke Registries (CNSRs) I and III.

Cumulative incidence rates of stroke recurrence are shown in Figure 1. The crude rates of stroke recurrence at 3, 6, and 12 months decreased significantly, from 1410 individuals (12.9%), 1748 individuals (16.0%), and 1939 individuals (17.7%) during 2007 to 2008 to 664 individuals (6.4%; *P* < .001), 839 individuals (8.1%; *P* < < .001), and 1044 individuals (10.1%; *P* < .001) in 2015 to 2018 (Figure 1A). After adjusting for age, sex, and NIHSS score on admission, decreases in adjusted recurrence rates at 3, 6, and 12 months from CNSR I to III remained (10.8% [95% CI, 10.2%-11.4%]; 13.6% [95% CI, 13.0%-14.2%]; and 15.5% [95% CI, 14.8%-16.2%] to 8.6% [95% CI, 8.1%-9.1%]; *P* < .001; 10.7% [95% CI, 10.1%-11.3%]; *P* < .001; and 12.5% [95% CI, 11.9%-13.1%]; *P* < .001), for a decrease of 19.4% in rates at 12 months (Figure 1B).

### Changes in Performance of Secondary Preventive Medicine

**Figure 2.**
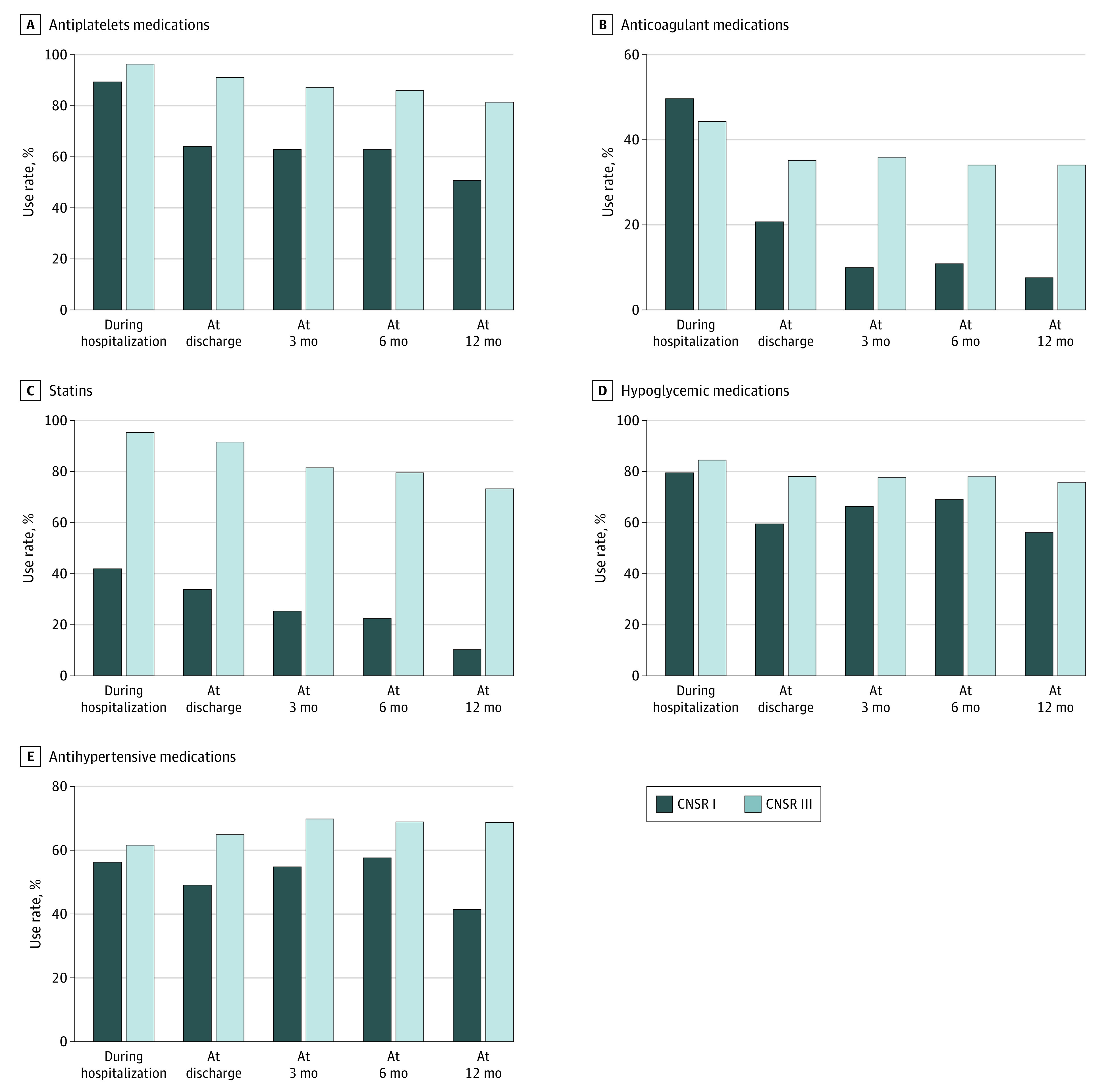
Performance of Secondary Prevention Medicines CNSR indicates China National Stroke Registry.

Given changes in stroke recurrence rates and risk factor patterns over 10 years, we also investigated performance of secondary prevention measures during hospitalization, at discharge, and 3, 6 and 12 months after discharge. As shown in Figure 2, rates and persistence of all types of secondary preventive medicine use increased between 2007 to 2008 and 2015 to 2018. The 12-month persistence was highest for hypoglycemic (1019 of 1788 individuals [57.0%]), followed by antiplatelet (4317 of 8391 individuals [51.5%]), antihypertensive (2268 of 5407 individuals [42.0%]), statin (923 of 8391 individuals [11.0%]), and anticoagulant (40 of 498 individuals [8.0%]) medications in 2007 to 2008. For 2015 to 2018, 12-month persistence was highest for antiplatelet (8144 of 9906 individuals [82.2%]), followed by hypoglycemic (1822 of 2380 individuals [76.6%]), statin (7330 of 9904 individuals [74.0%]), antihypertensive (4317 of 6228 individuals [69.3%]), and anticoagulant (232 of 672 individuals [34.5%]) medications.

### Changes in Risk Factor Patterns

**Figure 3.**
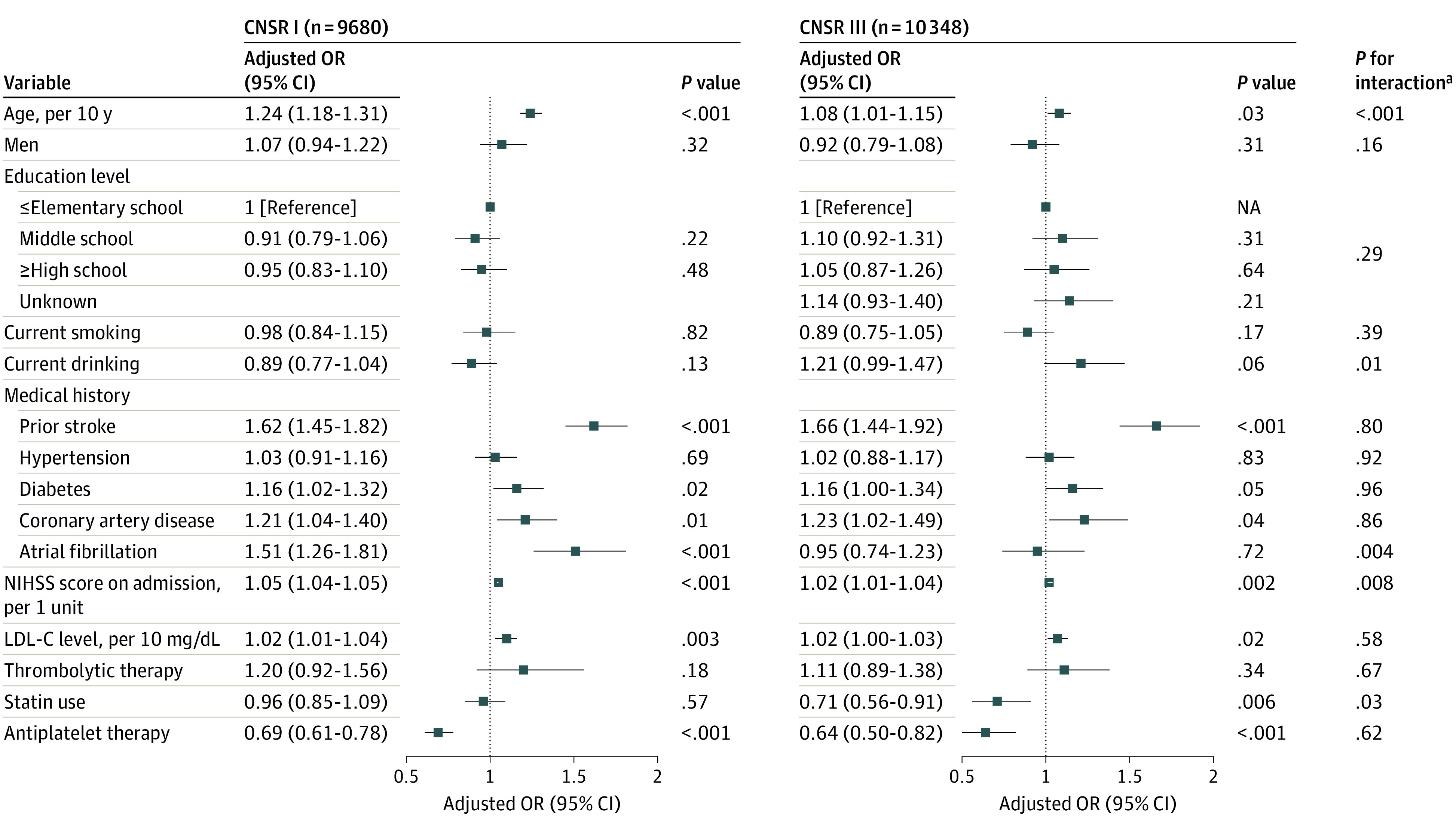
Logistic Regression Models of Factors Associated With 12-Month Stroke Recurrence Models examined interaction of study period (ie, China National Stroke Registry [CNSR] III vs I) with the association of demographic characteristics, risk factors, and treatments with 12-month stroke recurrence. To convert milligrams per deciliter to millimoles per liter, multiply by 0.0259. LDL-C indicates low-density lipoprotein cholesterol; NIHSS, National Institutes of Health Stroke Scale; OR, odds ratio. ^a^Test for interaction terms of study period and each covariate in the whole analysis set combined data from CNSR I and III.

In CNSR I, several factors were associated with increased risk of stroke recurrence, including age (OR per 10 years, 1.24; 95% CI, 1.18-1.31), prior stroke (OR, 1.62; 95% CI, 1.45-1.82), coronary heart disease (OR, 1.21; 95% CI, 1.04-1.40), atrial fibrillation (OR, 1.51; 95% CI, 1.26-1.81), NIHSS score (OR per 1 unit, 1.05; 95% CI, 1.04-1.05), and LDL-C level (OR per 10 mg/dL [0.259 mmol/L], 1.02; 95% CI, 1.01-1.04), while antiplatelet therapy was associated with decreased risk (OR, 0.69; 95% CI, 0.61-0.78). After 10 years, age (OR per 10 years, 1.08; 95% CI, 1.01-1.15), prior stroke (OR, 1.66; 95% CI, 1.44-1.92), coronary heart disease (OR, 1.23; 95% CI, 1.02-1.49), NIHSS score (OR per 1 unit, 1.02; 95% CI, 1.01-1.04), and LDL-C level (OR per 10 mg/dL, 1.02; 95% CI, 1.00-1.03) were still associated with increased risk of stroke recurrence. However, atrial fibrillation (OR, 0.95; 95% CI, 0.74-1.23) was no longer an independent risk factor associated with stroke recurrence in 2015 to 2018. Antiplatelet therapy remained a factor associated with protection against recurrence after 10 years (OR, 0.64; 95% CI, 0.50-0.82), and statin use became associated with protection (OR, 0.71; 95% CI, 0.56-0.91). In the whole analysis set, there were interactions of study period (ie, CNSR III vs I) with the association of age, atrial fibrillation, NIHSS score on admission, and statin use with stroke recurrence (Figure 3).

### Sensitivity Analysis

The model based on CNSR I using imputed LDL-C data is presented in eFigure 2 in the Supplement, and the results were consistent with those in Figure 3. Logistic regression models of patients from 33 hospitals that participated in CNSR I and III are presented in eTable 1 in the Supplement. Factors associated with stroke recurrence in CNSR I (ie, age, prior stroke, coronary heart disease, NIHSS score, and antiplatelet therapy) were mostly consistent with the main results; however, only prior stroke and NIHSS score were found to be associated with stroke recurrence in CNSR III.

Considering the competing risk for death, we excluded 12-month death from our logistic regression models (eTable 2 in the Supplement). Results were consistent with main results.

## Discussion

In this population-based cohort study spanning 10 years, we found that the rate of stroke recurrence at 12 months decreased by 19.4% between 2007 to 2008 and 2015 to 2018. Atrial fibrillation was not an independent risk factor for stroke recurrence in 2015 to 2018, whereas higher LDL-C levels were associated with increased stroke recurrence risk.

This study provides data on the risk of stroke recurrence in Chinese patients with IS. We assessed the dynamics of the stroke recurrence rate based on large-scale nationwide registries in China and observed a significant decrease in 12-month recurrence rate, from 15.5% to 12.5%, in the past decade. Our findings were similar to data from the Taiwan National Health Insurance program, which found that the rate of 12-month stroke recurrence decreased by 18% from 2000 to 2011 among 291 381 patients experiencing their first IS,^24^ supporting significant progress in stroke management. The significant decrease in stroke recurrence may be largely associated with advances in use of and adherence to secondary preventive treatments in the past decade, especially antithrombotic drugs and statins.^25,26^ As shown in this study, rates of secondary preventive medicine use have increased markedly in the past decade, including rates of medicine prescription at discharge and compliance with secondary preventive therapies. However, other factors could not be ruled out given that there were large differences in baseline characteristics of patients in CNSR I vs CNSR III, in that patients in CNSR III were younger and had lower stroke severity and lower lipid levels. Although we adjusted for age, sex, and NIHSS score in the recurrence rate analysis, the association of changes in demographics and other factors with the results cannot be ruled out. Owing to the heterogeneity of hospitals in CNSR I and III, we performed a sensitivity analysis of patient data from 33 hospitals but failed to find meaningful changes in risk factor patterns compared with those in the primary analysis. However, this may be associated with use of hospitals that were of a high grade and provided high-quality care, with tertiary hospitals accounting for approximately 91% of included hospitals. They may therefore not be nationally representative.

This study found that antiplatelet treatment was still associated with decreased risk of stroke recurrence at 10 years, suggesting that the current use of antiplatelets may require further optimization, such as administering dual antiplatelet agents to eligible patients. The role of antiplatelet therapy in secondary stroke prevention has been well established since 1997,^27,28^ but the rate of antiplatelet use increased significantly only after the findings of the Clopidogrel With Aspirin in Acute Minor Stroke or Transient Ischemic Attack (CHANCE) study^29^ provided further evidence of the effectiveness of antiplatelets. Our results also showed that 12-month persistence of anticoagulants increased from 8.0% in 2007 to 2008 to 34.5% in 2015 to 2018, which is consistent with a prior report^30^ of oral anticoagulant use based on the Chinese Stroke Center Alliance. This advancement in the use of anticoagulants may explain why atrial fibrillation was no longer an independent risk factor associated with stroke recurrence in 2015 to 2018. Additionally, we found that LDL-C level remained a risk factor associated with stroke recurrence in 2015 to 2018 and that statin use remained a factor associated with protection against stroke recurrence, despite increased levels of prescription and compliance rates for statins and hypoglycemics. We speculate that this may be associated with inadequate control of LDL-C levels. First, medication use is still suboptimal, especially that of statins. Prior data based on the Stroke Prevention by Aggressive Reduction in Cholesterol Levels trial^31,32^ supported use of intensive lipid-lowering therapy to reduce risk of atherosclerotic vascular disease, but guidelines for intensive statins for the acute management of IS were advanced relatively slowly.^5,33,34^ These updates of guidelines and increases in research evidence have provided more references for the development of statin strategies for patients with IS, but a certain evidence-practice gap remains.^35^ Second, other mechanisms may exist through which LDL-C levels are associated with the stroke process.^36,37^ Further studies are needed to clarify this issue.

### Limitations

Several limitations of this study must be considered. First, owing to the lack of data on blood markers in CNSR I, we were unable to investigate changes in patterns of novel marker risk factors, such as inflammatory biomarker, associated with stroke recurrence. Second, while multivariable regression models used in the study controlled for confounders, collider bias may have been introduced at the same time. Effect sizes of the associations between risk factors and stroke recurrence estimated from the study need further studies to validate. Third, we used serial cross-sectional data, and further research is needed to investigate any causal relationships between risk factors and stroke recurrence. Fourth, our analysis was based on a Chinese population, and the findings may not be generalizable to other populations.

## Conclusions

This cohort study found a decrease in stroke recurrence in China over the past decade; however, 12.5% of patients still experienced stroke recurrence within 12 months. Despite advancements in secondary prevention, high LDL-C levels, as well as age, prior stroke, and coronary heart disease, were associated with recurrence. Our findings suggest that programs and interventions to intensively control risk factors, including LDL-C levels, may be needed to further reduce stroke recurrence.

## Notes

Supplement.
eMethods.
**eTable 1.** Sensitivity Analysis on Patients From 33 Hospitals Included in China National Stroke Registry (CNSR) I and III **eTable 2.** Sensitivity Analysis With Death Excluded **eFigure 1.** Flowchart of Patient Selection **eFigure 2.** Regression Model of Factors Associated With Stroke Recurrence Using Imputed Low-Density Lipoprotein Cholesterol (LDL-C) Data
eReferences.
