# Supplementary material for: Trends and Risk Factors Associated With Stroke Recurrence in China, 2007-2018
Source: JAMA Netw Open. 2022 Jun 15;5(6):e2216341. doi: 10.1001/jamanetworkopen.2022.16341 (PMC9201671; doi:10.1001/jamanetworkopen.2022.16341)
Supplement: Supplement. — eMethods. eTable 1. Sensitivity Analysis on Patients From 33 Hospitals Included in China National Stroke Registry (CNSR) I and III eTable 2. Sensitivity Analysis With Death Excluded eFigure 1. Flowchart of Patient Selection eFigure 2. Regression Model of Factors Associated With Stroke Recurrence Using Imputed Low-Density Lipoprotein Cholesterol (LDL-C) Data eReferences. [file jamanetwopen-e2216341-s001.pdf]

Supplemental Online Content

Xu J, Zhang X, Jin A, et al. Trends and risk factors associated with stroke recurrence in China, 2007-2018. *JAMA Netw Open*. 2022;5(6):e2216341. doi:10.1001/jamanetworkopen.2022.16341

eMethods.
eTable 1. Sensitivity Analysis on Patients From 33 Hospitals Included in China National Stroke Registry (CNSR) I and III
eTable 2. Sensitivity Analysis With Death Excluded
eFigure 1. Flowchart of Patient Selection
eFigure 2. Regression Model of Factors Associated With Stroke Recurrence Using Imputed Low-Density Lipoprotein Cholesterol (LDL-C) Data
eReferences.

This supplemental material has been provided by the authors to give readers additional information about their work.

© 2022 Xu J et al. *JAMA Network Open.*

eMethods

Definitions of variables

Hypertension was defined as a self-reported history of hypertension, or taking oral antihypertension drugs, or documented to have hypertension in hospital admissions (blood pressure of 140/90 mmHg or higher). Diabetes mellitus was defined as a self-reported history of diabetes mellitus, or taking oral hypoglycemic agents/insulin. Coronary artery disease was defined as a self-reported history of coronary artery disease, or confirmed to have coronary artery disease or coronary artery disease by a cardiologist in hospital admissions. Atrial fibrillation was defined as a history of atrial fibrillation, confirmed by at least one electrocardiogram, or the presence of the arrhythmia during hospitalization.

We focused on 5 evidence-based secondary preventive interventions, including antiplatelet, statins, anticoagulant, antihypertensive, and antidiabetic. (1) Antiplatelet therapy was given to patients with no contraindications. (2) Anticoagulation was administered if the patient had a clear indication (presumed cardiac source of embolus, such as atrial fibrillation or prosthetic cardiac valve). (3) Antihypertensive therapy was given to those with hypertension and with no contraindications. (4) Antidiabetic therapy was given to those with diabetes mellitus and with no contraindications. (5) As for the use of statins, both the CNSR I and CNSR III studies were conducted after publication of the SPARCL study(1), when the use of statins was gradually proposed and applied in acute ischemic cerebrovascular events. Statins were given to patients with no contraindications.

Information on hospitals of study patients

	Hospitals, No. (%)		
	132 hospitals included in CNSR I	164 hospitals included in CNSR III	33 hospitals included both in CNSR I and CNSR III
Hospital type			
Grade II	32 (24.2)	31 (18.9)	3 (9.1)
Grade III	100 (75.8)	133 (81.1)	30 (90.9)
Region			
East	79 (59.8)	98 (59.8)	22 (66.7)
Central	32 (24.2)	56 (34.1)	7 (21.2)
West	21 (15.9)	10 (6.1)	4 (12.1)
Economic zones			
Northeast	20 (15.2)	11 (6.7)	4 (12.1)
North China	32 (24.2)	54 (32.9)	9 (27.3)
Eastern China	35 (26.5)	47 (28.7)	12 (36.4)
Northwest	13 (9.8)	6 (3.7)	3 (9.1)

© 2022 Xu J et al. *JAMA Network Open.*

Southwest	8 (6.1)	4 (2.4)	1 (3.0)
Central and Southern China	24 (18.2))	42 (14.6)	4 (12.1)
Infrastructures			
Number of beds in total*	800 (150, 2,500)	1,255 (500, 2,000)	1,600 (750, 3,200)
Number of beds in ND	45 (20, 176)	107 (40, 280)	104 (40, 280)
Stroke out-patients clinic	112 (84.8)	140 (85.4)	27 (81.8)
Emergency department	81 (61.4)	164 (100)	33 (100)
NICU	78 (59.1)	113 (68.9)	28 (84.8)
Stroke Unit	39 (29.5)	126 (76.8)	28 (84.8)
Inpatients' rehabilitation	92 (69.7))	162 (98.8)	33 (100)
Personnel			
Neurological rehabilitators	97 (73.5)	145 (88.4))	28 (84.8)
Speech therapist	52 (39.4)	124 (75.6)	22 (66.7)
Physiotherapist	45 (34.1)	158 (96.3)	32 (97.0)
Diagnostic procedure			
Brain CT scan	85 (64.4)	164 (100)	33 (100)
Digital subtraction angiography	112 (84.8)	150 (91.5)	32 (97.0)
Extracranial duplex sonography	121 (91.7)	158 (96.3)	29 (87.9)

*median with the 5th-95th percentile.

Abbreviation: CNSR I= the China National Stroke Registry; CNSR III= the Third China National Stroke Registry; ND= Neurology Department; NICU= Neurological Intensive Care Unit

List of the medications in each category

	In CNSR I	In CNSR III
Antiplatelets		
	Aspirin	Aspirin
	Clopidogrel	Clopidogrel
	Dipyrid amole	Dipyrid amole
	Ticlopidine	Cilostazol
	Aspirin/Dipyrid amole	Ozagrel
	GP IIb/IIIa receptor antagonist	Others
Anticoagulants		
	Warfarin	Warfarin
	Low molecular heparin	Low molecular heparin

© 2022 Xu J et al. *JAMA Network Open.*

	Heparin	Heparin
	Others	Rivaroxaban
		Dabigatran
		Apixaban
		Others
Hypoglycemics		
	Insulin	Insulin
	Biguanides	Biguanides
	Sulfonylureas	Sulfonylureas
	Alpha glycosidase inhibitor	Alpha glycosidase inhibitor
	Others	Thiazolidinediones
		Glinides
		Others
Antihypertensives		
	Calcium ion antagonist	Calcium ion antagonist
	Angiotensin converting enzyme inhibitor	Angiotensin converting enzyme inhibitor
	Diuretic	Angiotensin II receptor antagonist
	Beta blockers	Diuretic
	Alpha blockers	Beta blockers
		Alpha blockers
		Alpha/beta blockers
		Others
Statins		
	/*	Atorvastatin
		Rosuvastatin
		Simvastatin
		Pravastatin
		Lovastatin
		Fluvastatin
		Pitavastatin

* The information regarding the specific categories of statins was not collected in CNSR I.

© 2022 Xu J et al. *JAMA Network Open.*

eTable 1. Sensitivity Analysis on Patients From 33 Hospitals Included in China National Stroke Registry (CNSR) I and III

Variables	Stroke recurrence in 12 months			
	CNSR I, n=2,233		CNSR III, n=2,723	
	Adjusted OR (95% CI)	P value	Adjusted OR (95% CI)	p value
Age, per 10 years	1.20 (1.08-1.34)	0.001	1.07 (0.95-1.22)	0.28
Men	0.96 (0.74-1.25)	0.76	0.82 (0.60-1.13)	0.23
Education level				
≤Elementary school	Ref.		Ref.	
Middle school	0.90 (0.67-1.22)	0.51	1.10 (0.76-1.59)	0.62
≥High school	0.91 (0.67-1.23)	0.53	1.01 (0.69-1.47)	0.97
Unknown			0.92 (0.61-1.38)	0.67
Current smoking	0.79 (0.56-1.10)	0.16	0.88 (0.62-1.23)	0.45
Current drinking	0.93 (0.67-1.27)	0.64	1.24 (0.85-1.81)	0.26
Medical History				
Prior stroke	1.69 (1.34-2.14)	<0.001	1.56 (1.16-2.09)	0.003
Hypertension	1.12 (0.88-1.42)	0.37	1.14 (0.85-1.51)	0.39
Diabetes	1.15 (0.88-1.50)	0.31	1.26 (0.95-1.67)	0.12
Coronary artery disease	1.41 (1.06-1.91)	0.02	1.03 (0.68-1.55)	0.90
Atrial fibrillation	1.05 (0.70-1.56)	0.82	0.94 (0.56-1.59)	0.82
NIHSS score on admission, per 1 unit	1.05 (1.03-1.06)	<0.001	1.03 (1.003-1.06)	0.03
LDL-C level, per 10 mg/dL	1.02 (0.99-1.05)	0.23	1.003 (0.97-1.04)	0.86
Thrombolytic therapy	1.11 (0.67-1.84)	0.69	0.99 (0.58-1.67)	0.97
Statin use	1.08 (0.84-1.40)	0.55	0.79 (0.50-1.26)	0.32
Antiplatelet therapy	0.59 (0.45-0.76)	<0.001	0.83 (0.52-1.33)	0.44

Abbreviation: CNSR I= the China National Stroke Registry; CNSR III= the Third China National Stroke Registry; OR= odds ratio; CI= confidence interval; NIHSS=National Institutes of Health Stroke Scale; LDL-C=low density lipoprotein cholesterol.

© 2022 Xu J et al. *JAMA Network Open.*

eTable 2. Sensitivity Analysis With Death Excluded

Variables	Stroke recurrence in 12 months			
	CNSR I, n=8,389		CNSR III, n=10,014	
	Adjusted OR (95% CI)	p value	Adjusted OR (95% CI)	p value
Age, per 10 years	1.03 (0.96-1.10)	0.37	1.06 (0.99-1.14)	0.08
Men	1.13 (0.95-1.34)	0.17	0.89 (0.76-1.06)	0.19
Education level				
≤Elementary school	Ref.		Ref.	
Middle school	1.02 (0.85-1.22)	0.88	1.12 (0.93-1.36)	0.24
≥High school	1.08 (0.90-1.29)	0.41	1.11 (0.91-1.35)	0.30
Unknown			1.22 (0.98-1.52)	0.08
Current smoking	0.94 (0.78-1.13)	0.48	0.88 (0.74-1.06)	0.18
Current drinking	0.90 (0.75-1.08)	0.25	1.23 (1.002-1.51)	0.05
Medical History				
Prior stroke	1.65 (1.43-1.91)	<0.001	1.62 (1.39-1.89)	<0.001
Hypertension	1.14 (0.97-1.33)	0.11	1.05 (0.90-1.21)	0.55
Diabetes	1.22 (1.03-1.43)	0.02	1.17 (1.01-1.37)	0.04
Coronary artery disease	1.50 (1.25-1.81)	<0.001	1.24 (1.01-1.53)	0.04
Atrial fibrillation	1.44 (1.10-1.87)	0.007	0.91 (0.68-1.21)	0.51
NIHSS score on admission, per 1 unit	1.004 (0.99-1.02)	0.51	1.01 (0.997-1.03)	0.12
LDL-C level, per 10 mg/dL	1.01 (0.99-1.03)	0.25	1.02 (1.005-1.04)	0.01
Thrombolytic therapy	1.67 (1.22-2.29)	0.001	1.16 (0.92-1.45)	0.22
Statin use	1.04 (0.89-1.21)	0.66	0.84 (0.54-1.10)	0.21
Antiplatelet therapy	0.90 (0.77-1.07)	0.23	0.69 (0.53-0.90)	0.007

Abbreviation: CNSR I= the China National Stroke Registry; CNSR III= the Third China National Stroke Registry; OR= odds ratio; CI= confidence interval; NIHSS=National Institutes of Health Stroke Scale; LDL-C=low density lipoprotein cholesterol.

© 2022 Xu J et al. *JAMA Network Open.*

eFigure 1. Flowchart of Patient Selection

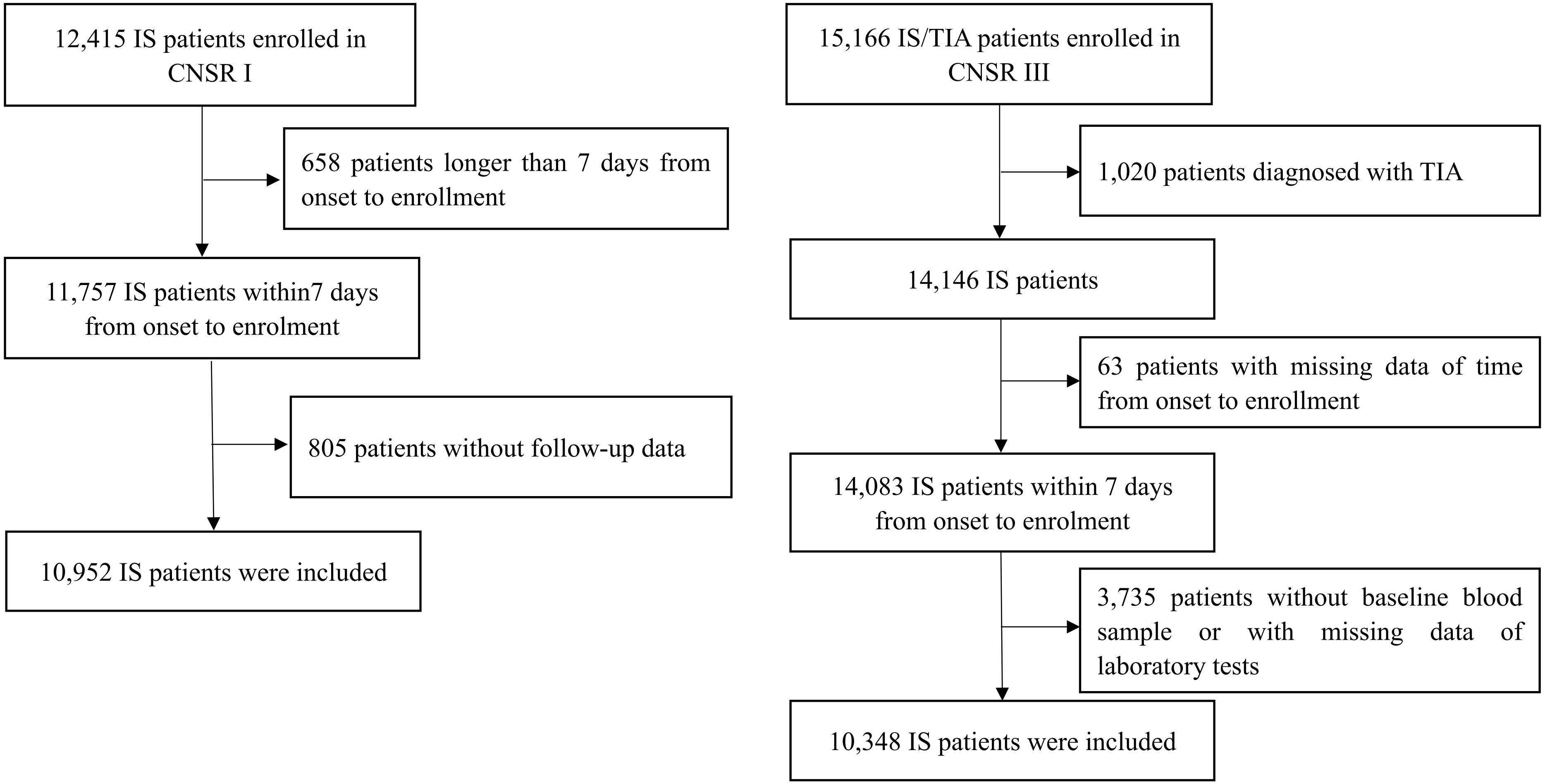

© 2022 Xu J et al. *JAMA Network Open.*

eFigure 2. Regression Model of Factors Associated With Stroke Recurrence Using Imputed Low-Density Lipoprotein Cholesterol (LDL-C) Data

Variables	CNSR I (n=1,0952) Adjusted OR (95%CI)	p value
Age, per 10 years	1.23 (1.17-1.29)	<0.001
Men	1.04 (0.98-1.11)	0.25
Education level		
≤Elementary school	Ref.	
Middle school	0.97 (0.89-1.05)	0.52
≥High school	0.98 (0.90-1.06)	0.55
Unknown		
Current smoking	0.99 (0.86-1.14)	0.94
Current drinking	0.86 (0.75-0.99)	0.04
Medical History		
Prior stroke	1.58 (1.42-1.76)	<0.001
Hypertension	1.00 (0.90-1.12)	0.99
Diabetes	1.18 (1.05-1.33)	0.009
Coronary artery disease	1.16 (1.01-1.33)	0.04
Atrial fibrillation	1.46 (1.23-1.73)	<0.001
NIHSS score on admission, per 1 unit	1.04 (1.03-1.05)	<0.001
LDL-C level, per 10 mg/dL	1.02 (1.01-1.04)	0.009
Thrombolytic therapy	0.98 (0.90-1.06)	0.70
Statin use	0.96 (0.85-1.08)	0.50
Antiplatelet therapy	0.70 (0.62-0.79)	<0.001

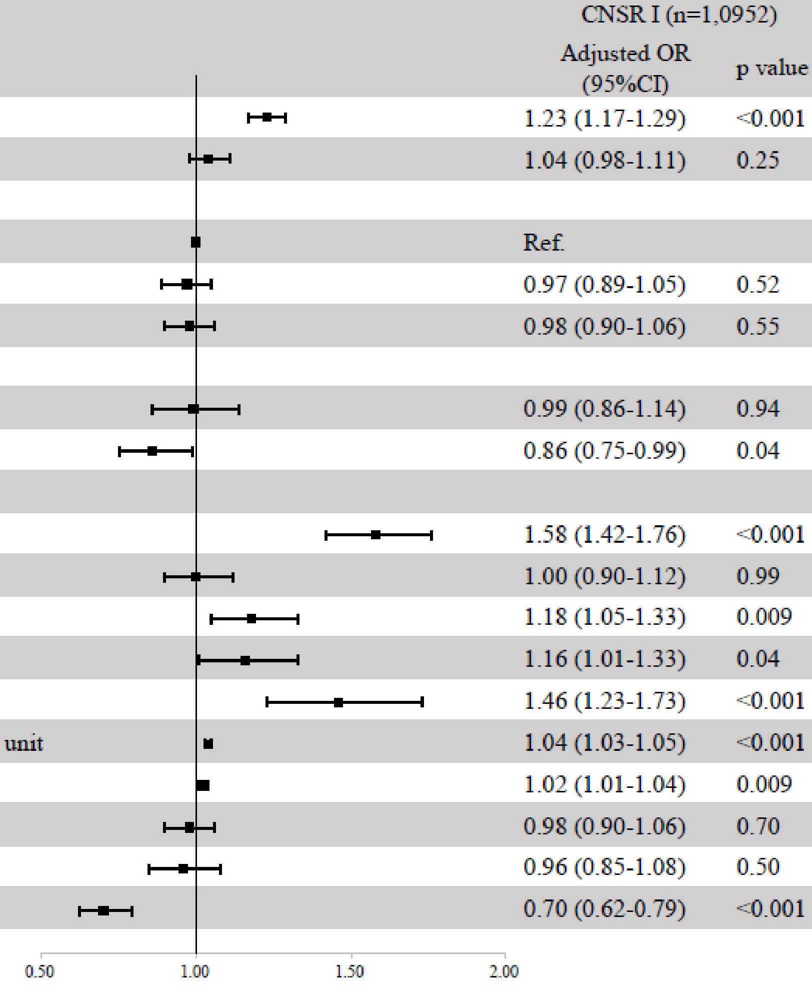

© 2022 Xu J et al. *JAMA Network Open.*

eReferences

1. Amarenco P, Bogousslavsky J, Callahan A, 3rd, Goldstein LB, Hennerici M, Rudolph AE, et al. High-dose atorvastatin after stroke or transient ischemic attack. The New England journal of medicine. 2006;355(6):549-59.

© 2022 Xu J et al. *JAMA Network Open.*
